# Magnetic Reconnection in Space: An Introduction

**DOI:** 10.1007/s11214-025-01145-x

**Published:** 2025-02-12

**Authors:** J. L. Burch, Rumi Nakamura

**Affiliations:** 1 Southwest Research Institute, San Antonio, TX USA; 2 Space Research Institute, Austrian Academy of Sciences, Graz, Austria; 3 International Space Science Institute, Bern, Switzerland

## Abstract

An International Space Science Institute (ISSI) workshop was convened to assess recent rapid advances in studies of magnetic reconnection made possible by the NASA Magnetospheric Multiscale (MMS) mission and to place them in context with concurrent advances in solar physics by the Parker Solar Probe, astrophysics, planetary science and laboratory plasma physics. The review papers resulting from this study focus primarily on results obtained by MMS, and these papers are complemented by reports of advances in magnetic reconnection physics in these other plasma environments. This paper introduces the topical collection “Magnetic Reconnection: Explosive Energy Conversion in Space Plasmas”, in particular introducing the new capabilities of the MMS mission used in majority of the articles in the collection and briefly summarizing the advances obtained from MMS.

## Context and Purpose

The study of magnetic reconnection is a relatively young branch of plasma physics, which has recently garnered great interest as its importance in the laboratory, the Earth’s magnetosphere, the solar photosphere and corona and objects such as accretion disks surrounding neutron stars and black holes and supernova remnants has become widely recognized. The common occurrence of magnetic reconnection and its frequent explosive nature make it one of the most important agents of energy transfer throughout the universe. A recent commentary by Hesse and Cassak (2020) describes the universal importance of reconnection along with prospects for its further understanding.

An International Space Science Institute (ISSI) workshop was convened to assess recent rapid advances in this field made possible by the NASA Magnetospheric Multiscale (MMS) mission (Burch et al. 2016a) and to place them in context with concurrent advances in solar physics by the Parker Solar Probe (Drake et al. 2025, this collection), astrophysics (Guo et al. 2024, this collection), planetary science (Gershman et al. 2024, this collection) and laboratory plasma physics (Ji et al. 2023b, this collection). The review papers resulting from this study focus to a large extent on the results obtained by MMS, and these papers are complemented by reports of advances in magnetic reconnection physics in these other plasma environments.

Leading up to the launch of MMS in March 2015, significant progress in understanding magnetic reconnection at the MHD and ion scales was made by the European Space Agency Cluster mission (e.g., Eastwood et al. 2010) while theoretical predictions of electron-scale phenomena by plasma simulations helped set the stage for future measurements (e.g., Hesse et al. 2014; Bessho et al. 2014). As described by Burch and Drake (2009), four great mysteries of magnetic reconnection were: (1) What produces dissipation in a collisionless plasma, allowing reconnection to occur? (2) What determines the aspect ratio of the dissipation region and the rate of release of magnetic energy? (3) What is the “spark” that causes the magnetic energy that has built up over a period of time to be suddenly released? and (4) What is the mechanism for efficient conversion of magnetic energy into the kinetic energy of charged particles? The unprecedented temporal and spatial resolution of the MMS measurements (Burch et al. 2016a) have led to significant progress toward solving these four mysteries while at the same time revealing many unanticipated aspects of magnetic reconnection in the boundary regions of the Earth’s magnetosphere. Note that the above mysteries of magnetic reconnection apply to many other plasma environments as discussed in Ji et al. (2023a). Outstanding questions of magnetic reconnection in different plasma environments are further discussed in Nakamura et al. (2025, this collection).

## New Capabilities of MMS

By 2005 MMS had become the next NASA Solar-Terrestrial Probe. This event followed the ever-increasing scientific focus on magnetic reconnection that had occurred in the previous 25 years. The International Sun-Earth Explorer mission (ISEE) had made the first measurement of the reconnection ion exhaust at the dayside magnetopause (Paschmann et al. 1979), thereby establishing the existence of magnetic reconnection as an important mechanism of energy-transfer from the solar wind to the Earth’s magnetosphere. Subsequent investigations by the WIND, Polar, Cluster and THEMIS missions formed a comprehensive picture of how magnetic reconnection and other associated plasma processes operate at the fluid and ion scales in the boundary regions of the magnetosphere (e.g., Øieroset et al. 2001; Mozer et al. 2002; Eastwood et al. 2010; Angelopoulos et al. 2008). The new frontier for MMS was recognized to be the electron diffusion region, where magnetic fields in adjacent regions (magnetosheath-magnetosphere, north-south tail lobes) become interlinked. The primary advance that was needed was to increase the measurement cadence of the three-dimensional electron distribution function by at least a factor of 100. This requirement was reached through a simple consideration of the following estimates that were made at the time: The electron diffusion region has a width of a few km; the magnetopause moves radially inward and outward at speeds of a few 10s of km/s; and at least three measurements of the distribution function within the diffusion region are needed to characterize it. These numbers led to a round-figure minimum time resolution for electrons of 30 ms. Because of the larger size of the ion diffusion region, the time resolution for ions was relaxed to 150 ms. This major advance in time resolution required a new approach to measuring plasmas over 4$\boldsymbol{\pi}$ steradians (Pollock et al. 2016). Most previous missions used the spacecraft rotation to sample the full sky with a 2D angular array of sensors, but the required time resolution for MMS led to spacecraft rotation speeds that were not technically achievable. The new approach taken by MMS was to use multiple 2D arrays (tophats) with ±22.5° electrostatic deflection of the azimuthal fields of view. This approach required accurate intercalibration of eight electron and eight ion instruments on each spacecraft and across the four spacecraft.

Other key requirements were (1) to determine the reconnection electric field with high-resolution three-axis electric field measurements, which had been difficult or impossible to achieve in the previous missions (Torbert et al. 2016a; Ergun et al. 2016); (2) to measure plasma composition accurately in the presence of the high proton fluxes at the magnetopause, which required a new instrument design (Burch et al. 2005; Young et al. 2016); (3) to measure energetic electrons and ions produced by magnetic reconnection and associated processes (Mauk et al. 2016); (4) to control the spacecraft potential to <4 V, as was done on Cluster (Torkar et al. 2016); and (5) to maintain a tetrahedron of four spacecraft with separations in the range from 10 to 160 km for dayside and nightside observations of reconnection (Tooley et al. 2016). The measurements made by the four MMS spacecraft are summarized in Table 1.

**Table 1:** Summary of measurements made on each MMS spacecraft.

Measurement	Time Resolution	Sensitivity/Energy Resolution
DC B	3D to ∼1 ms	<0.1 nT
DC E	3D to ∼1 ms	<0.5 mV/m
	SC Potential (proxy for $n_{e}$) to ∼1 ms	
AC B	Waveform, spectra to 6 kHz	<2 × 10^−5^ nT/Hz^1/2^ @ 1 kHz
AC E	Waveform, spectra to 100 kHz	<1 × 10^7^ V/m-Hz^1/2^ @ 10 kHz
Plasma Electrons	3D $f_{e}(v)$ every 30 ms; 1 eV to 30 keV	20% ΔE/E resolution
	Electron beams at single E to 1 ms	Poisson Statistics limited
Plasma Ions	3D $f_{i}(v)$ every 150 ms; 1 eV to 20 keV/q	20% ΔE/E resolution
		Poisson Statistics limited
Plasma Ion	3D $f_{i}(v)$ every 10 s	20% ΔE/E resolution
Composition	10 eV/q to 30 keV/q for H^+^, He^++^, He^+^, O^+^	
Energetic Ions/Electrons	3D *f*(*v*) every 10 s; 20 keV to 1000 keV	35% ΔE/E resolution
Energetic Ions	3D $f_{i}(v)$ every 20 s; 40 keV to 1000 keV	35% ΔE/E resolution
	For H^+^, He^++^, He^+^, O^+^	

Calibrated Level-2 data in physical units are provided to the public in common data format (CDF) with 30-day latency through the MMS Science Data Center at https://lasp.colorado.edu/mms/sdc/public/. To aid in MMS data plotting and analysis, an extensive library of IDL and Python codes are maintained in the Space Physics Environment Data Analysis System (SPEDAS) (Angelopoulos et al. 2019).

## Summary of Results from MMS

Regarding reconnection mystery (1), the plasma simulations of Hesse et al. (2014) suggested that accelerated electrons could rapidly carry energy from the magnetic field out of the diffusion region from laminar reconnection without the need for turbulence or the associated anomalous resistivity. Early MMS measurements (Burch et al. 2016b) confirmed the existence of the crescent-type distributions predicted by Hesse et al. (2014) and by Bessho et al. (2014) while also confirming the prediction of Cassak and Shay (2007) that the dissipation occurs Earthward of the X line for asymmetric reconnection at the magnetopause. Torbert et al. (2016b) showed further that the non-ideal electric field in generalized Ohm’s law was produced mainly by the divergence of the electron pressure tensor with a much smaller contribution from electron inertia and a significant residual, which could represent anomalous resistivity. The primary conclusion from these early studies is that at the magnetopause the reconnection electric field is produced by non-isotropic electron pressure, that the electron crescent distributions carry the out-of-plane current and the dissipation, as measured by $\mathbf{J}\cdot\mathbf{E}$, is caused completely by the electron dynamics. Norgren et al. (2025, this collection) provide a detailed discussion of electron dynamics in the electron diffusion region (EDR), while further results on the generalized Ohm’s Law and reconnection rate from theory and measurements are reviewed by Liu et al. (2025, this collection).

As noted by Burch and Drake (2009), there is the possibility that “with turbulence, the field lines would be strongly twisted so that multiple ones could reconnect simultaneously, vastly increasing the reconnection rate.” The Earth’s magnetosheath is a region that is often turbulent by virtue of the dynamical interaction of solar-wind plasma with the bow shock. While reconnecting magnetosheath current sheets have been observed by Cluster (Retino et al. 2007) and THEMIS (Øieroset et al. 2017), the electron dynamics were not observable owing to their 4-s and 3-s time resolutions, respectively. With MMS, turbulent reconnection is observed routinely in the magnetosheath at the electron scale with the significant result that often there is only an electron diffusion region, which is not embedded within an ion diffusion region as occurs in the standard reconnection model (Phan et al. 2018).

In the MMS era, the magnetosheath has become an important laboratory for the study of kinetic plasma turbulence and its relationship with magnetic reconnection (Wilder et al. 2017; Chasapis et al. 2018; Bandyopadhyay et al. 2021). Stawarz et al. (2024, this collection) cover the latest advances in turbulent reconnection by MMS in the magnetosheath and bow shock as well as in Kelvin-Helmholtz vortices in the flank magnetopause. In the magnetosheath, MMS revealed that reconnection between interlinked flux tubes originating from multiple X-lines leads to the formation of flux ropes or flux transfer events (FTEs). This 3D FTE formation process occurs predominantly when the interplanetary magnetic field has a strong east-west component, a condition that leads magnetic field lines to collide and reconnect. These phenomena are covered in detail by Hwang et al. (2023, this collection).

Regarding reconnection mystery (2), the rate of release of magnetic energy, or the reconnection rate, can be measured by determining the aspect ratio of the electron diffusion region, by measuring the inflow rate of electrons into the diffusion region, and by measuring the reconnection electric field. Theoretical estimates summarized by Cassak et al. (2017) are that the normalized reconnection rate is near 0.1. MMS data have been used for all three approaches with the aspect ratio being determined for a magnetotail reconnection event by Nakamura et al. (2019); the electron inflow velocity being measured by Burch et al. (2020, 2022); and the reconnection electric field being determined by Nakamura et al. (2018) and Genestreti et al. (2018). These measurements have yielded normalized reconnection rates in the range of 0.05 to 0.25 with the highest value being for the Phan et al. (2018) electron-only reconnection event. Further discussion of the latest theoretical and experimental advances on the reconnection rate appears in Liu et al. (2025, this collection).

For reconnection mystery (3), the magnetotail is an opportune region for determining what initiates reconnection because there is a growth phase during which the magnetic flux is transferred from the dayside to the nightside magnetosphere leading eventually to the rapid onset of magnetic reconnection across the tail neutral sheet and the initiation of a magnetospheric substorm (Angelopoulos et al. 2008). A recent result by Genestreti et al. (2023) showed how a thinning magnetotail current sheet triggered by a solar-wind pressure pulse develops multiple X lines with low reconnection rates until one of them reaches lobe field lines. At this time, rapid reconnection is initiated, a primary X line is formed, and the other X lines are swept down the tail within the exhaust of the primary X line. This observation of the initiation of rapid reconnection and its implications are discussed further by Nakamura et al. (2025, this collection).

Mystery (4) stems from the observation of electrons and ions with energies of hundreds of keV during magnetotail reconnection events. These energies exceed the full potential drop across the magnetotail and far exceed the energies that can result from the reconnection electric field of a few mV/m or the energy of reconnection outflows, which proceed at near the Alfvén speed. While some energetic particles, particularly on the day side of the magnetosphere can be explained by leakage of radiation-belt particles through the magnetopause, this process does not apply in the mid to distant magnetotail. Betatron and Fermi acceleration, along with acceleration by parallel electric fields have been predicted and observed by MMS to be important in the Earthward flows, reconnection exhaust and separatrices, respectively (Turner et al. 2016; Ma et al. 2022). Particularly intriguing is the turbulent acceleration of ions and electrons near the reconnection X line and the surrounding diffusion region (Ergun et al. 2022). These results are discussed in detail by Oka et al. (2023, this collection).

An important and unexpected result from MMS is the ubiquitous occurrence of reconnection wherever thin current sheets co-exist with either laminar or turbulent magnetic-field reversals. Examples include thin current sheets embedded within the vortices of Kelvin-Helmholtz MHD instability, which often occur along the flanks of the magnetotail (Eriksson et al. 2016; Hwang et al. 2023, this collection); and the bow shock, which also has been found by MMS to contain reconnecting thin current sheets on a regular basis (Stawarz et al. 2024, this collection).

The reconnection diffusion region has been found to be the site of a wide variety of plasma waves with frequencies from below the ion cyclotron frequency to above the electron plasma frequency, which often develop from plasma instabilities that result from the accumulation of free energy. Advances from MMS concerning these waves and the instabilities that cause them are reviewed by Graham et al. (2025, this collection).

During the time over which rapid advances in reconnection physics have been made by MMS, similar advances have been made in the plasma simulations that predicted many of the experimental results and were later used to provide deeper theoretical understanding of unexpected measurements. The development of plasma simulations during the MMS era is summarized by Shay et al. (2025, this collection). Important advances have also been made in methods for analyzing the multi-spacecraft MMS data including reconstruction of the magnetic-field surrounding reconnection sites. A comprehensive review of these analysis methods is provided by Hasegawa et al. (2024, this collection).

## Advances in Theory and Modeling

As reviewed by Shay et al. (2025, this collection), a wide variety of plasma simulation techniques have been developed to explore the kinetic physics of reconnection diffusion regions, the transfer of particles and energy to the region closely surrounding the diffusion regions, as well as the macroscale and global effects of reconnection. Because of limitations on computer speed and memory, complete simulation of kinetic physics in the diffusion region is still not feasible at realistic ion-to-electron mass ratios or with time-resolution sufficient to track electron-scale wave phenomena. However, evolution of computer capabilities is steadily improving this situation.

Before the launch of MMS, plasma simulation was somewhat more advanced than the measurements because they could access electron kinetic scales with the most common technique being 2.5-D particle-in-cell (PIC) simulations. With this technique electric and magnetic fields and plasma distribution functions are resolved in 3D but are only allowed to vary along a 2D grid. While limited in scope, these simulations nevertheless made important predictions, some of which have been verified with MMS data. A classic example is the electron crescent distribution, which is described by Genestreti et al. (2025, this collection).

As noted by Shay et al. (2025, this collection), mesoscale, macroscale and up to global simulation can be handled by embedding PIC codes into MHD codes. A similar approach is used in solar flare physics where explicit particle-acceleration codes are embedded in MHD codes as described by Drake et al. (2025, this collection).

## Summary and Conclusions

Considering the many advances in magnetic reconnection physics that have been made by MMS, it is possible to identify further advances that can be made by this remarkable mission or by subsequent missions that may focus on specific targets for further study. These potential advances are discussed in the outlook paper by Nakamura et al. (2025, this collection). One example is the investigation of cross-scale coupling through which the many microscale reconnection phenomena identified by MMS propagate to the successively larger meso and macro scales. Early attempts at understanding the first stages of cross-scale coupling are being made by MMS through the use of asymmetric tetrahedron and string-of-pearls formations. Ultimately, the determination of how magnetic reconnection affects global magnetospheric dynamics will require coordinated measurements at the various scales from micro to global. Such a large undertaking would no doubt require significant multi-national cooperation, which would be well justified based on the extensive advances made at the microscale by MMS. Outstanding questions about the coupling of the MMS-observed kinetic processes and large-scale magnetospheric processes are reviewed by Fuselier et al. (2024, this collection).
